# A Survey of Antimicrobial Resistance Determinants in Category A Select Agents, Exempt Strains, and Near-Neighbor Species

**DOI:** 10.3390/ijms21051669

**Published:** 2020-02-29

**Authors:** Chris R. Taitt, Tomasz A. Leski, Amy Chen, Kimberly L. Berk, Robert W. Dorsey, Michael J. Gregory, Shanmuga Sozhamannan, Kenneth G. Frey, Diane L. Dutt, Gary J. Vora

**Affiliations:** 1 US Naval Research Laboratory, Center for Biomolecular Science & Engineering, Washington, DC 20375, USA; tomasz.leski@nrl.navy.mil (T.A.L.); gary.vora@nrl.navy.mil (G.J.V.); 2 Karle’s Fellow, US Naval Research Laboratory, Washington, DC 20375, USA; ajchenac@gmail.com; 3 US Army Combat Capabilities Development Command (CCDC) Chemical Biological Center, Edgewood, MD 21010, USA; Kimberly.l.berk.civ@mail.mil (K.L.B.); Robert.w.dorsey.civ@mail.mil (R.W.D.); 4 US Naval Medical Research Center - Biological Defense Research Directorate, Frederick, MD 20910, USAkenneth.g.frey4.civ@mail.mil (K.G.F.); 5 Defense Biological Product Assurance Office, Joint Program Executive Office for Chemical, Biological, Radiological and Nuclear Defense (CBRND), Frederick, MD 21702, USA; shanmuga.sozhamannan.ctr@mail.mil; 6 Logistics Management Institute, Tysons, VA 22012, USA; 7 Defense Threat Reduction Agency, Joint Science and Technology Office, Ft. Belvoir, VA 22060, USA; diane.l.dutt.civ@mail.mil

**Keywords:** Antimicrobial resistance, *Yersinia* spp., *Francisella* spp., *Bacillus* spp., microarrays, high resolution melt analysis (HRMA), quinolone resistance determining regions (QRDRs)

## Abstract

A dramatic increase in global antimicrobial resistance (AMR) has been well documented. Of particular concern is the dearth of information regarding the spectrum and prevalence of AMR within Category A Select Agents. Here, we performed a survey of horizontally and vertically transferred AMR determinants among Category A agents and their near neighbors. Microarrays provided broad spectrum screening of 127 *Francisella* spp., *Yersinia* spp., and *Bacillus* spp. strains for the presence/absence of 500+ AMR genes (or families of genes). Detecting a broad variety of AMR genes in each genus, microarray analysis also picked up the presence of an engineered plasmid in a *Y. pestis* strain. High resolution melt analysis (HRMA) was also used to assess the presence of quinolone resistance-associated mutations in 100 of these strains. Though HRMA was able to detect resistance-causing point mutations in *B. anthracis* strains, it was not capable of discriminating these point mutations from other nucleotide substitutions (e.g., arising from sequence differences in near neighbors). Though these technologies are well-established, to our knowledge, this is the largest survey of Category A agents and their near-neighbor species for genes covering multiple mechanisms of AMR.

## 1. Introduction

Ever-increasing levels of antimicrobial resistance (AMR) are considered a top global health concern and pose a “threat to the success and continuation of clinical medicine as we know it.” [1,2]. The issue of AMR is also challenging for biosecurity reasons. While morbidity/mortality and ease of dissemination/weaponization remain the top reasons for classification of various species as Category A threat agents, increasingly greater attention is being paid to the potential for multidrug resistance (MDR) within these strains. Whether naturally occurring or engineered, the emergence and spread of agents expressing multidrug resistance (MDR) present a clear risk for significant consequences [3]. Even without deliberate engineering of MDR into threat strains, there exists considerable risk of the emergence and spread of resistant strains. Rapid identification of virulent strains and AMR profiles is essential for implementation of suitable infection control measures.

Standard therapeutic guidelines for treatment of infections with Category A agents are well defined (Table 1). Of particular concern, resistance to fluoroquinolones—among the top choices for treating anthrax, tularemia, and plague (Table 1)—is commonly associated with point mutations in DNA gyrase (*gyrA*, *gyrB*) and topoisomerase IV (*parC*, *parE*) genes. Mutations within the quinolone resistance determining regions (QRDRs) of these genes can occur spontaneously or with intentional application of selective pressure [4,5,6,7,8,9].

Horizontally transferred resistance determinants also pose a significant threat. MDR *Yersinia pestis* strains isolated in Madagascar in the 1990s harbored plasmids with up to eight AMR determinants, at least four of which confer resistance to recommended therapeutics or prophylactic treatments [13,14,15,16,17]. Fortunately, the strains responsible for more recent outbreaks in Madagascar, the Democratic Republic of Congo, and Uganda do not carry these plasmids and are susceptible to the standard therapeutics [18,19]. Even so, AMR determinants present on mobile elements have the potential to spread widely; a number of reports have described the means of transferring naturally occurring plasmids harboring AMR determinants to and from these strains [14,20,21]. Furthermore, ever increasing numbers of cloning vectors are being described that are capable of being transferred to and from *Y. pestis* and other Category A agents, such as *Bacillus anthracis* and *Francisella tularensis* [22,23,24,25,26]. In most cases, common AMR markers are used for selection and could potentially be suggestive of engineered strains.

In spite of the large number of publications describing the pathophysiology of these Category A agents, there still remains a dearth of information regarding the full spectrum and prevalence of AMR and their molecular mechanisms within these agents and their near neighbors. For this reason, we performed a survey of 62 *Bacillus* spp., 23 *Francisella* spp., and 42 *Yersinia* spp. strains for both horizontally and vertically transferred AMR determinants; included amongst these strains were 46 Category A Select Agents. Microarrays were used for broad spectrum screening of all 127 strains for the presence/absence of 500+ AMR genes and gene families, while high resolution melt analysis (HRMA) was used to assess the presence of quinolone resistance-associated mutations in 100 of these strains. To our knowledge, this is the largest survey of Category A agents and their near-neighbor species for genes covering multiple mechanisms of AMR.

## 2. Results and Discussion

Phenotypic antimicrobial susceptibility testing (AST) is still considered the gold standard for determination of resistance profiles. However, ASTs cannot be used to trace the spread of AMR strains nor the mobile genetic elements responsible for transmission of resistance. Phenotypic testing further cannot distinguish between various resistance mechanisms with overlapping specificities and are generally inadequate for epidemiological and forensic tracking. While not always predictive of phenotype, molecular tests can nonetheless determine the genetic basis and overall mechanisms for resistance, as well as the potential for transmission to other strains or species via horizontal gene transfer.

Here, we genetically characterized 127 archived Category A agents and their near neighbors with respect to AMR. To accomplish this, we determined the presence/absence of >500 AMR determinants using the ARDM v.3.1. In parallel, we used a combination of targeted PCR and HRMA to identify mutations in *gyrA*, *parC*, and *parE* that confer resistance to fluoroquinolones used as first-line therapeutics. By combining the broad screening capability of microarrays with the fine discriminatory capabilities of HRMA, we detected markers acquired through both horizontal transfer and mutation. The dataset generated here provides a baseline measurement of the spectrum and prevalence of various resistance mechanisms in both Category A select agents and their near neighbors. These baseline data may be helpful in development of predictive algorithms designed to assess risk associated with outbreaks with naturally occurring or engineered threats, as well as in guiding development of new therapeutics.

### 2.1. Testing for Horizontally-Acquired Genes—Microarray Analysis

**Figure 1:**
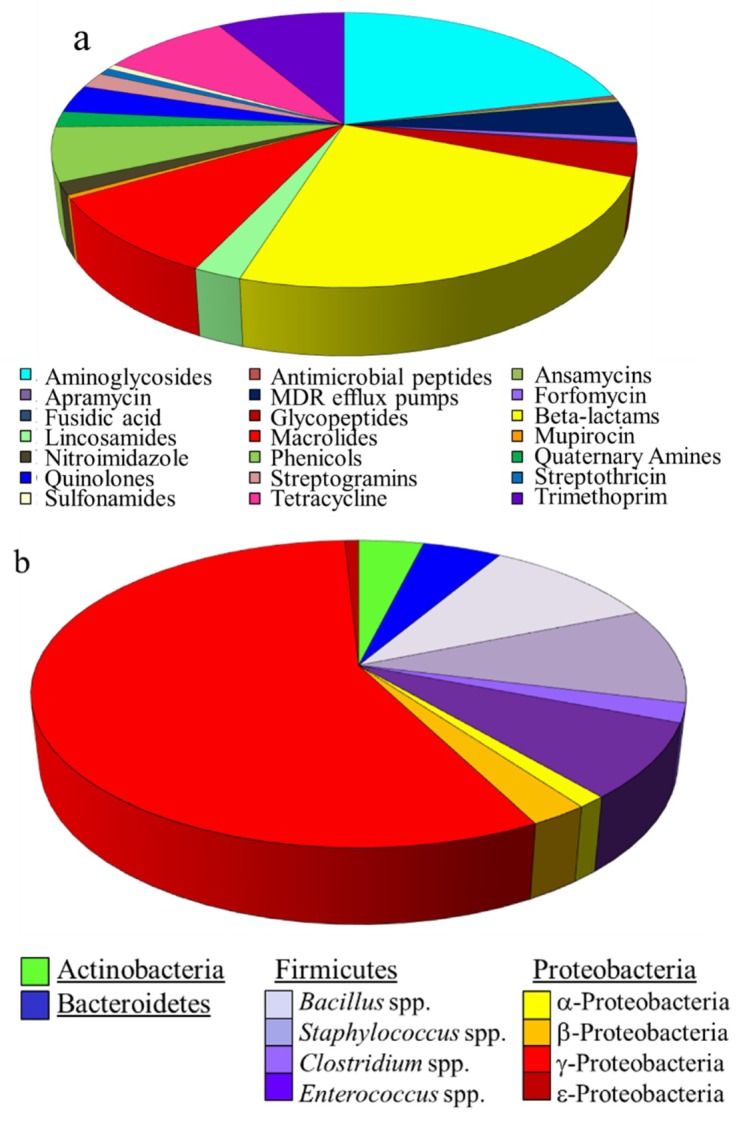
Content of the ARDM v.3.1: (**a**) Categories of antimicrobials for which resistance determinants are included in the ARDM v.3.1; (**b**) Bacterial sources of resistance determinants.

Based on the ARDM v.2 [27] and v.3 [28], the ARDM v.3.1 contains probes directed against emerging AMR determinants of global importance (e.g., *bla*_NDM_), as well as those derived from several Select Agent species (including *F. tularensis*, *B. anthracis*, *Y. pestis*), their near neighbors, and more distantly related species in the same genera. In total, the ARDM v.3.1 content (4480 probes per two sub-arrays) covers 512 AMR determinants, conferring resistance to 19 categories of antimicrobials (Figure 1a). Microarray content was derived from *Actinobacteria*, *Firmicutes*, *Bacteroidetes*, and *Proteobacteria* phyla (Figure 1b). Pertinent to the current study, the following determinants were derived from Select Agents and their near neighbors: 7 genes from *Francisella* spp. (*blaA*, *blaB*, *tet*, *bcr/cfl1*, *bcr/cflA2*, and *bcr/cflA3* from *F. tularensis*, and *bla* from *F. philomiragia*); 6 genes from *Yersinia* spp. (*qacE*, *yegB*, *emrA*, *emrB*, and *bacA* from *Y. pestis* CO92, and *vatF/sat* from *Y. enterocolitica*); and 30 genes from a wide variety of *Bacillus* species (including *bla1*, *bla2, bla*[Vollum1], *bla*[Vollum3], *bla*[Vollum4], *aacC7*, *aad*[Vollum1], *aad*[Vollum2], *aph*[Vollum1], *aph*[Vollum2], *aph*[Vollum3], *mph*, *tetM*, *vanY*, *cat* from *B. anthracis*; and *fosB* from *B. cereus*).

#### 2.1.1. Francisella spp.

Twenty *F. tularensis* and three *F. philomiragia* strains were characterized on the ARDM v.3.1. Not surprisingly, all *F. tularensis* strains were positive for the six *F. tularensis*-derived genes and the three *F. philomiragia* strains were positive for the single *F. philomiragia*-derived gene, *bla* (Table 2). One of the *F. philomiragia* strains, Jensen O#319-029, was also positive for the presence of *bcr/cflA1*. The reference sequence for *bcr/cflA1* was compared with the published sequence for this strain (CP009343.1) and the other two *F. philomiragia* strains via BLAST. The *bcr/cflA1* reference sequence had 91.6% sequence identity with Jensen O#319-029 while sequence identity with Jensen O#319L and Jensen O#319-036 was only ~65%. Similar BLAST comparisons showed ~80% or lower sequence identities between the three *F. philomiragia* strains and the other *F. tularensis*-derived genes on the microarray. Though not PCR-confirmed, these results support previous observations [27,29] that the specificity of the ARDM can enable detection of closely related genes (or families of genes), but that a sequence identity of >90% is required.

#### 2.1.2. Yersinia spp.

DNA from 42 *Yersinia* spp. strains was characterized on the ARDM v.3.1. Strains included 26 isolates from the closely related *Y. pestis*/*Y. pseudotuberculosis* group [30], seven *Y. enterocolitica* strains, and nine strains from six species related more distantly to both *Y. pestis*/*Y. pseudotuberculosis* and *Y*. *enterocolitica*. Not surprisingly, all of the *Y. pestis* (17 isolates) and *Y. pseudotuberculosis* (9 isolates) strains were positive for the same five *Y. pestis*-derived genes: *bacA*, *qacE*, *emrA*, *emrB*, and *yegB*. Importantly, *Y. pestis* KIM(pCD1Ap+) was also positive for the presence of *bla*_TEM_; KIM(pCD1Ap+) is a KIM5 strain harboring plasmid pCD1 into which *bla*_TEM_ was inserted into the *yadA* gene (i.e., as a selectable marker) [31]. Therefore, the ARDM was able to accurately detect the presence of a non-native AMR gene engineered into *Y. pestis*.

All *Y. enterocolitica* strains except strain 8081 were positive for the *Y. enterocolitica*-derived gene, *vatF/sat* (Table 3); strain 8081 tested negative for this gene. A BLAST search of *Y. enterocolitica* 8081 whole genome sequence for *vatF/sat* yielded a single sequence with only 79% identity, indicating that the microarray’s negative results were indeed accurate. None of the more distantly related *Yersinia* spp. strains were positive for any of the 512 resistance determinants on the ARDM v.3.1, including those derived from *Yersinia* spp. (Table 3).

#### 2.1.3. Bacillus spp.

**Figure 2:**
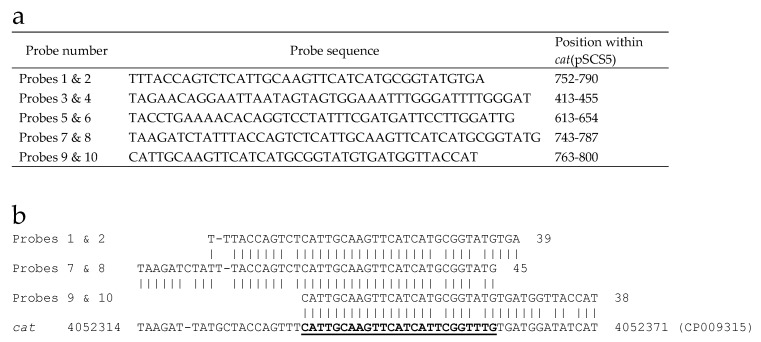
Source of false-positive calls for *cat*(pSCS5) in *B. anthracis*. (**a**) Sequences of *cat*(pSCS5) probes derived from *S. aureus* gene (accession no. M58515). (**b**) Alignment of probe sequences with *B. anthracis cat* gene (CP009315) represented on the ARDM v.3.1. The 25-nt sequence with 92% identity to the *B. anthracis cat* gene is underlined.

DNA preparations from 62 different strains from the *B. cereus* group were characterized on the microarray, including 41 *B. anthracis*, 13 *B. cereus*, and eight *B. thuringiensis* strains. Not surprisingly, all of the *B. anthracis* strains were positive for the same sixteen *B. anthracis*- or *B. cereus*-derived genes (Table 4). A plasmid-borne gene encoding a chloramphenicol acetyltransferase originally isolated from *Staphylococcus aureus*, c*at*(pSCS5), was also detected in a number of *B. anthracis* samples. The c*at*(pSCS5) gene has no identical allele in *B. anthracis.* However, six of the ARDM’s 10 probes for this gene have a 25-nt sequence with 92% identity to the *B. anthracis*-derived *cat* gene represented on the ARDM v.3.1 (Figure 2); this latter *cat* gene was observed in all *B. anthracis* and most *B. cereus* and *B. thuringiensis* strains. These *cat*(pSCS5)-positive calls therefore represent false-positive results. To minimize the potential for future false-positives, redesign of the ARDM—or more specifically, the group of c*at*(pSCS5) probes—is needed to improve specificity and eliminate redundant sequences.

**Figure 3:**
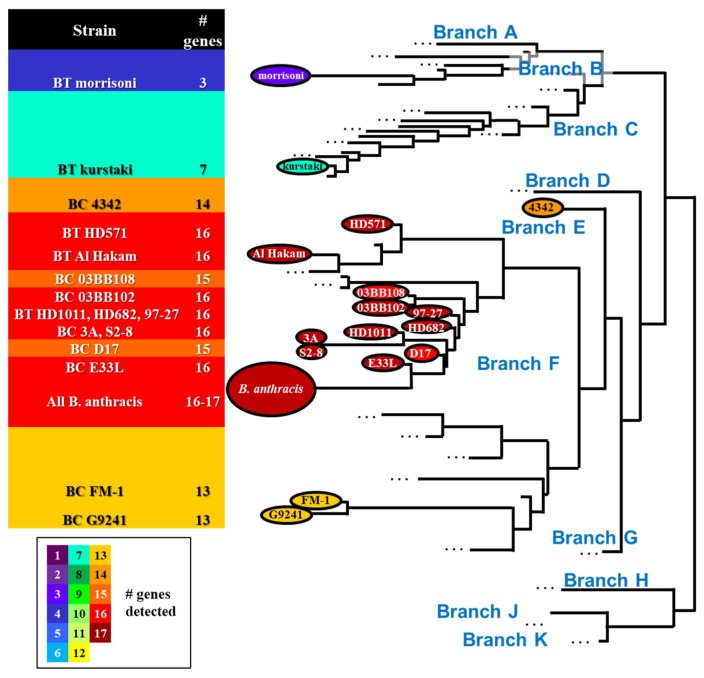
Phylogenetic tree of *B. cereus* group strains showing locations of *B. anthracis*, *B. cereus* (BC), and *B. thuringiensis* (BT) strains tested (tree based on [32,33]). The number of genes detected in *B. cereus* and *B. thuringiensis* strains is correlated to the phylogenetic distance from *B. anthracis*.

Four *B. cereus* and five *B. thuringiensis* were also positive for the same 16 genes observed in all of the *B. anthracis* strains. Of the remaining samples that did not harbor all 16 genes, the number of positive genes ranged from two (*B. thuringiensis* var. israelensis) to 15 genes (*B. cereus* D17 and 03BB108). Perhaps not surprisingly, the number of genes detected was generally correlated to the phylogenetic similarity between strains (Figure 3), with fewer genes detected in near-neighbor strains more distantly related to *B. anthracis*.

In general, the microarray-based survey of AMR determinants did not reveal any significant surprises, with Select Agent species harboring AMR genes expected to be present. Similarly, near-neighbor species harbored the expected genes (e.g., *F. philomiragia*-derived *bla* in all three *F. philomiragia* strains, *vatF/sat* in all but one *Y. enterocolitica* strains). The microarray was, however, able to detect a non-native AMR determinant (*bla*_TEM_) in a single *Y. pestis* strain (KIM (pCD1Ap)); this gene was engineered into a plasmid in the parent strain as a selectable marker in knock-out experiments. The ability to detect such a non-native gene in a Select Agent species—within <24-h and without *a prior*i knowledge of its potential presence—illustrates the value of a broad-based screening method such as microarray analysis.

### 2.2. HRMA

High-resolution melt analysis (HRMA) combines the finely detailed discriminatory capabilities of sequence analysis with the rapidity, relevance, and user-friendliness of PCR. Originally developed for genotyping studies, HRMA determines the melting profile of double stranded DNA sequences using a DNA intercalating dye. The shape and position of the melting curve yield information about the composition of a given DNA sequence from which mutational data can be derived; substitution of a G/C with A/T will reduce the T_m_ while an A/T→G/C change will result in a similar increase in T_m_. In this manner, HRMA can provide the capability to distinguish allelic variants which play an important role in resistance arising from mutations. As these mutations are not detectable with the ARDM alone, HRMA is a valuable approach that is highly complementary to the ARDM.

High level resistance to ciprofloxacin—a first- or second-line therapeutic for tularemia, plague, and anthrax—often arises from point mutations in the QRDRs of *gyrA*, *gyrB, parC*, *parE*. Loveless [34] previously demonstrated that HRMA could be used to detect point mutations in QRDRs of *F. tularensis*, *Y. pestis*, and *B. anthracis*. Here, we applied their strategy to assess sequence differences in 10 *Francisella* spp. strains (*gyrA*, *parE*), 34 *Yersinia* spp. strains (*gyrA* only), and 60 *Bacillus* spp. strains (*gyrA*, *parC*) to determine the practicality of this method for rapid screening.

#### 2.2.1. Francisella spp.

**Figure 4:**
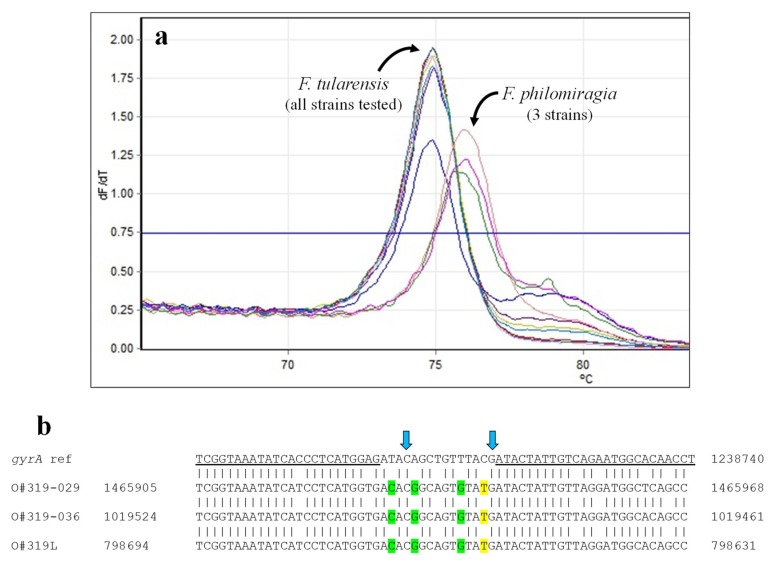
HRMA—*Francisella* spp. *gyrA*. (**a**) Melt curves for *Francisella* spp. *gyrA* amplicons. (**b**) Comparison of reference *gyrA* sequence with published sequences from *F. philomiragia* strains (see Table 9). All *F. tularensis* strains tested are identical to the reference sequence within the stretch interrogated. Primer sequences are underlined. Arrows indicate nucleotide positions where changes are demonstrated to result in quinolone resistance [34,35]. Sequence differences predicted to result in increased or decreased T_m_s are highlighted in green and yellow, respectively.

QRDRs in *gyrA* and *parC* were assessed by HRMA in seven *F. tularensis* and three *F. philomiragia* strains using PCR primers designed to bracket mutations observed to result in ciprofloxacin resistance [9,34,35]. In the *gyrA* assay, all *F. tularensis* strains clustered together (Table 5, Figure 4); these results are consistent with the phenotypic susceptibility of all strains for which ASTs have been reported [36]. Formation of a separate cluster by all three *F. philomiragia*, with T_m_s nearly a degree higher, was not surprising. There are three A/T→C/G and one C/G→A/T changes within the *gyrA* target sequence; these changes would be expected to—and did—cause a significant change in T_m_. Differences within the primer sequences were also likely responsible for the significantly higher C_t_s observed with the *F. philomiragia* samples (average C_t_ of 32.7 versus 13.8 for *F. tularensis*, *p* < 0.025).

**Figure 5:**
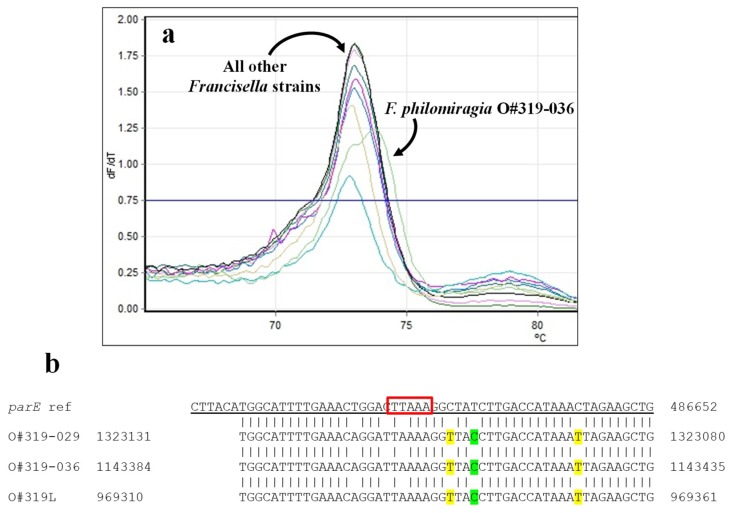
HRMA—*Francisella* spp. *parE*. (**a**) Melt curves for *Francisella* spp. *parE* amplicons. (**b**) Comparison of reference *parE* sequence with published sequences for the *F. philomiragia* strains (see Table 9). All *F. tularensis* strains tested are 98% identical to the reference sequence within this locus. Primer sequences are underlined. The sequence within the red box indicates the 5-nt deletion of interest [34]; mutations/substitutions predicted to increase or decrease amplicon T_m_s are highlighted in green or yellow, respectively.

The HRMA primers used here for the *parE* assay were designed to detect a 5-bp deletion previously noted by Loveless [34]. All of the strains—both *F. tularensis* and *F. philomiragia*—clustered together, in spite of second T_m_ peak observed with *F. philomiragia* O#319-036. Clustering together of the *F. tularensis* and *F. philomiragia* strains was not unexpected, though the clustering was not as tight as observed with *gyrA*. Though the 5-bp region interrogated had a single nucleotide difference (T→A) not predicted to cause a change in T_m_, there were multiple differences within the primer regions that might affect the T_m_s of half the amplicons in each PCR reaction (highlighted in Figure 5b). As with the *gyrA* amplifications, C_t_s for the three *F. philomiragia* strains were significantly greater than those of the *F. tularensis* strains (*p* < 0.005), likely due to these changes in the primed regions.

#### 2.2.2. Yersinia spp.

Multiple studies have reported ciprofloxacin resistance arising in *Y. pestis* from mutations in the *gyrA* QRDR [34,37,38,39,40], but to our knowledge, analogous mutations in *parC* or *parE* have not been documented. For this reason, only *gyrA*-specific HRMA assays were used with *Yersinia* spp. The site bracketed in the *Y. pestis* assay encompassed four mutations causing phenotypic ciprofloxacin resistance, with an additional two mutations found within the primer sequences [37,38].

**Figure 6:**
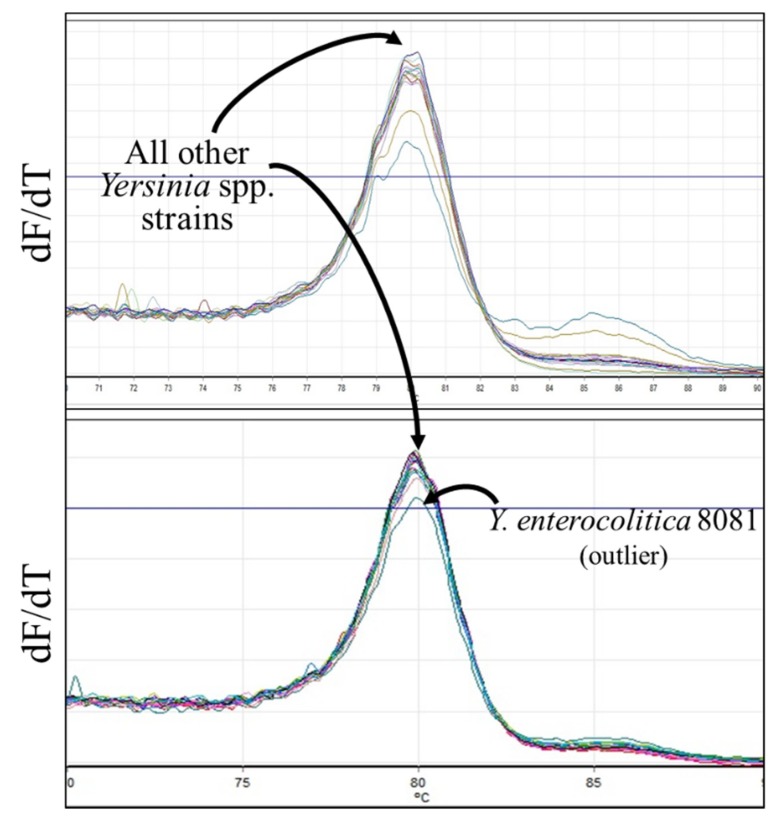
Melt curves for *Yersinia* spp. *gyrA*. All samples, independent of species, had melt curve profiles with T_m_s centered around 80.0 °C (panels show results obtained on different days). The melt curve for the outlier *Y. enterocolitica* 8081 is shown on the lower curve; there is no obvious feature responsible for its clustering outside of all other *Yersinia* spp. strains tested.

HRMA was performed on DNA from thirty-five *Yersinia* spp. strains, including 10 *Y. pestis*, eight *Y. pseudotuberculosis*, and seven *Y. enterocolitica* isolates. In many cases, two peaks (or a peak and a shoulder) were observed (Figure 6); T_m_s of the lower peaks ranged between 79.03 °C and 79.95 °C and of the higher peaks between 78.83 °C and 80.25 °C. All of the *Y. pestis* strains formed a single tight cluster with average T_m_ of 79.83; five *Y. pseudotuberculosis*, four *Y. enterocolitica*, and six more distantly related strains clustered with these *Y. pestis* strains (Table 6). A separate cluster was comprised of the remaining *Y. pseudotuberculosis* strains (AMC TB4, IP32953, strain 1), *Y. enterocolitica* DATR and WA, *Y. intermedia*, *Y. kristensenii*, and *Y. rohdei* strains. A single *Y. enterocolitica* isolate (strain 8081) was an outlier.

**Figure 7:**
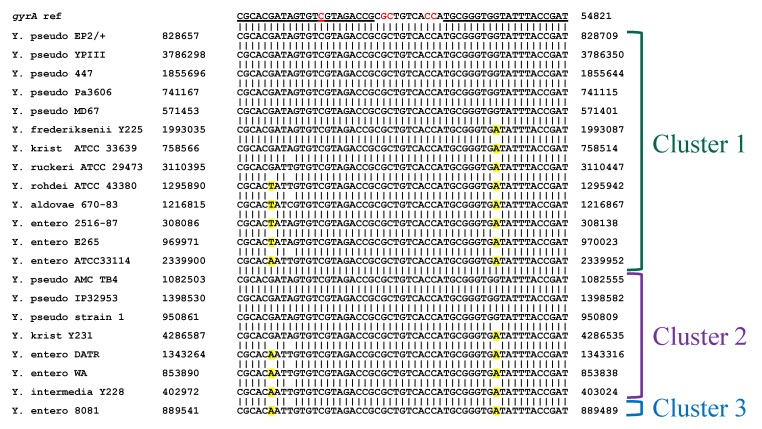
Alignment of interrogated *gyrA* sequences with published reference sequences from all non-*pestis Yersinia* spp. strains tested (see Table 11). The *Y. pestis* reference sequence is shown at the top with primer sequences underlined. Mutations at the positions shown in red have been demonstrated to cause quinolone resistance [37,38]. Sequence substitutions predicted to cause a decrease in T_m_ are highlighted in yellow. Y. entero = *Y. enterocolitica*; Y. krist = *Y. kristensenii*; Y. pseudo = *Y. pseudotuberculosis.*

Based on the published sequences available for the *Yersinia* spp. strains (Figure 7), the basis for principal component 1 used in the clustering analysis is unclear. Though all *Y. pestis* strains were placed in the same cluster, several *Y. pseudotuberculosis* strains with sequences identical to the reference sequence were placed in cluster 2. Similarly, amplicons from other strains having predicted C/G→A/T changes that would decrease T_m_ (typically within the primer regions) were split evenly between clusters 1 and 2. The predictability in clustering could potentially be improved through analysis of additional replicates. Furthermore, inclusion of quinolone-resistant strains with documented QRDR mutations (to which we did not have access) would help demonstrate the true efficacy in using HRMA to detect both species-specific and sequence-specific changes predictive of phenotype. As is, based on the current knowledge of known point mutations causing fluoroquinolone resistance and the location of sequence differences, none of the tested strains would be predicted to be resistant. Indeed, for those strains for which phenotypic susceptibility has been determined (Y. pestis Nairobi, PBM19, Pestoides F, Harbin35, Java9, CO92, Pestoides G, Angola, and Nicholisk 41), none are resistant [41]. At present, however, the utility of HRMA to detect such differences is not clear.

#### 2.2.3. Bacillus spp.

HRMA was performed on DNA preparations from 37 *B. anthracis* strains. This sample set included DNA preparations from both 18 wild-type strains and 19 plasmid-cured strains selected for development of high-level ciprofloxacin resistance [6] to assess whether documented mutations within *gyrA* and *parC* QRDRs could be detected.

**Figure 8:**
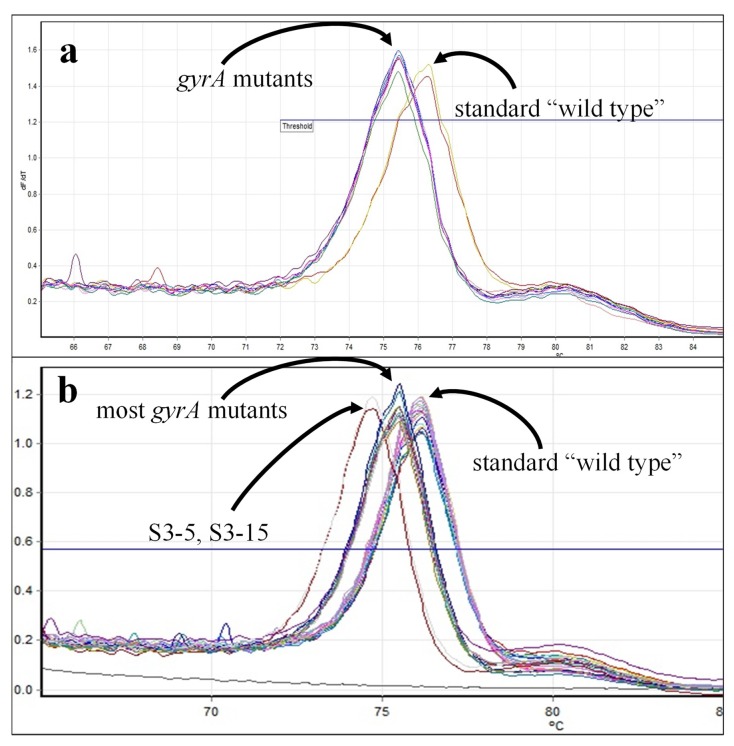
*GyrA* melt curves for *B. anthracis* strains. (**a**) Melt curves for wild-type strains, Sterne and ∆ANR, and *gyrA*-mutated ∆ANR strains, S1-1, S1-2, S2-1, S2-2, S2-3, S3-1, S3-3, and S3-4. (**b**) Melt curves for the remainder of wild-type and *gyrA*-mutated ∆ANR strains (see Table 7).

In HRMA for *gyrA*, all *B. anthracis* wild-type strains produced T_m_s in the range of 75.9 °C to 76.2 °C, whereas T_m_s for *B. anthracis gyrA* mutants had T_m_s at least 0.5 °C lower (Figure 8, Table 7). Principal component analysis yielded three clusters: wild-type strains (Cluster 1 = wt), Cluster 2 with most *gyrA*-mutant strains, and Cluster 3 comprising two samples with significantly lower T_m_s (mutant strains S3-5 and S3-15). Interestingly, strain S3-16 has two mutations in *gyrA* (C254→T and A266→C) that should thermodynamically balance each other; Loveless previously observed that this strain clustered with wild-type *B. anthracis* [34]. However, in our hands, this strain was found in Cluster 2 with other C/G→A/T-mutated *B. anthracis* strains (Table 7). Unpublished sequences of the targeted loci (S. Sozhamannan, C. Bernhards personal communication, manuscript in preparation) support clustering of the remaining mutants not previously described [6,34] away from wild-type *B. anthracis.*

HRMA and clustering of the same set of *B. anthracis* samples based on *parC* sequences were complicated by higher PCR failure rates and a narrower temperature range between T_m_s of wild-type and mutant amplicons. Furthermore, T_m_s for control strains could shift from day to day by as much as 0.5 °C, making it difficult to compare samples tested on different days, other than to indicate that they clustered with (or separate from) specific control samples (see footnote to Table 7).

**Figure 9:**
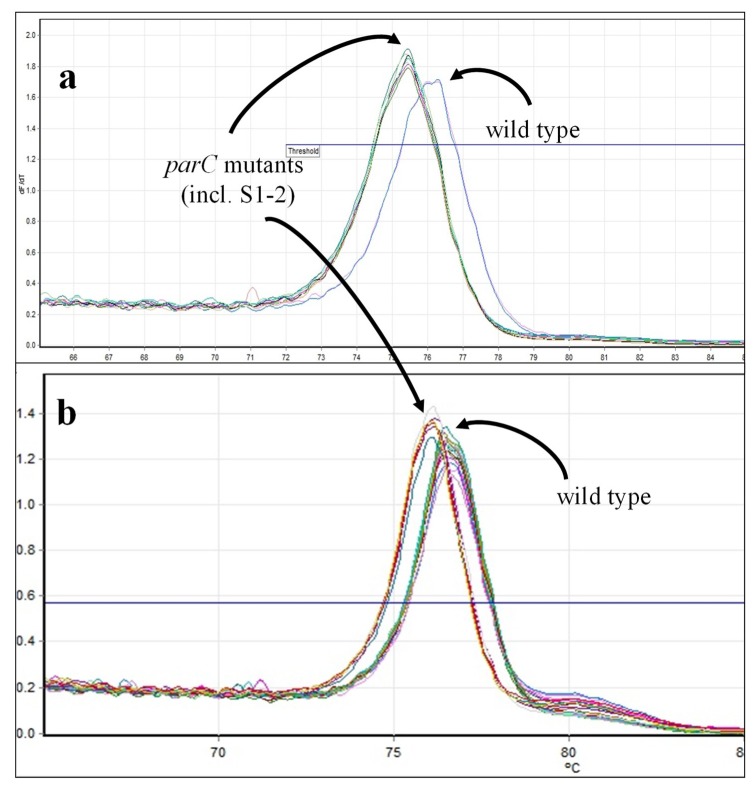
*ParC* melt curves for *B. anthracis* strains. (**a**) Melt curves for wild-type strains, Sterne and ∆ANR, and *gyrA*-mutated ΔANR strains, S1-1, S1-2, S2-1, S2-2, S2-3, S3-1, S3-3, and S3-4. (**b**) Melt curves for the remainder of wild-type and *gyrA*-mutated ΔANR strains (see Table 7).

Most of the *B. anthracis* strains with documented *parC* mutations clustered separately from wild-type *B. anthracis* (Figure 9; Table 7, Clusters 2A, 3A, 3B). Decreased T_m_s observed with mutant strains were consistent with C242→T changes. However, in contrast to previous observations [34], two strains that should have wild-type *parC* genes (S1-1 and S1-2 [6]) clustered separately from wild-type strains, with strain S1-2 as the sole member of Cluster 3A. From the melt curve, it is unclear why this sample clusters separately from all other strains tested. However, as with the *gyrA* results, unpublished sequences of the remaining mutants (S2-1 through S3-18) support their exclusion from the wild-type cluster (S. Sozhamannan, C. Bernhards, personal communication; manuscript in preparation).

Twelve *B. cereus* and eight *B. thuringiensis* strains were also analyzed by HRMA to determine whether their *gyrA* and *parC* sequences were sufficiently similar to *B. anthracis* to allow robust amplification, for species discrimination, and for detection of QRDR mutants.

**Figure 10:**
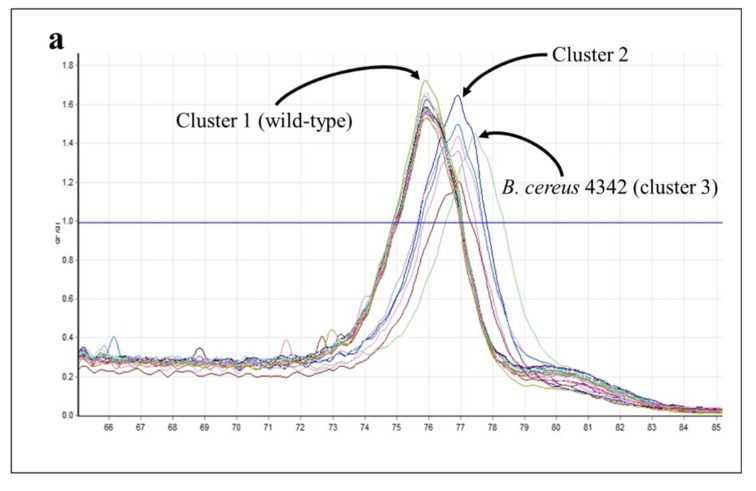

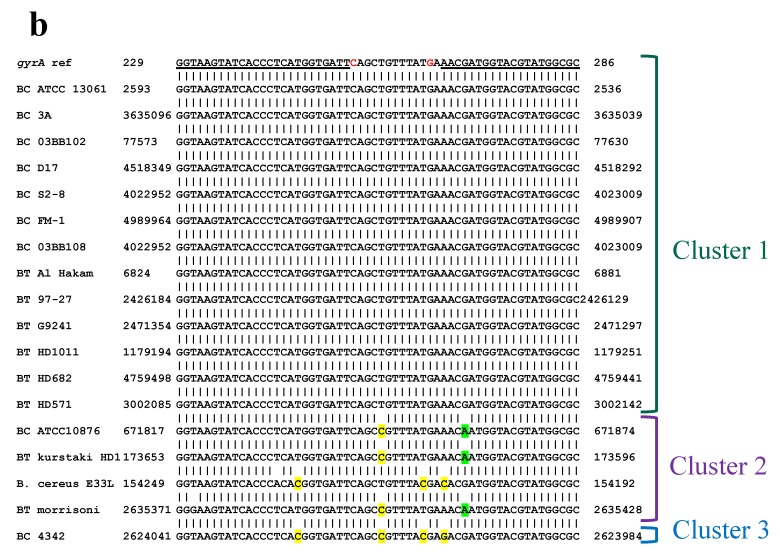
HRMA—*B. cereus* and *B. thuringiensis gyrA*. (**a**) Melt curves for *B. cereus* and *B. thuringiensis gyrA* amplicons. (**b**) Comparison of reference *gyrA* sequence with published sequences for the *B. cereus* (BC) and *B. thuringiensis* (BT) strains (see Table 13). Primer sequences are underlined; nucleotide substitutions at the locations shown in red have been documented to cause resistance. Substitutions predicted to increase or decrease amplicon T_m_s are highlighted in green or yellow, respectively.

Almost two-thirds (14/19) of the near neighbor strains clustered closely with wild-type *B. anthracis* (cluster 1) in *gyrA* assays (Table 8). Two additional clusters were comprised of five strains (cluster 2) and a single strain (cluster 3) with T_m_s that were 1–1.5 °C higher than the wild-type *B. anthracis* cluster (Figure 10). Cluster 2 represented four strains distantly related to *B. anthracis* (*B. thuringiensis* kurstaki, morrisoni, israelensis, and *B. cereus* ATCC 10876) and *B. cereus* E33L. Although closely related to *B. anthracis,* [42], E33L’s published sequence has, like other strains clustering with it, a T→C substitution within the interrogated sequence (Figure 10); this substitution—though in a location different from the others—would be expected to cause an increase in T_m_. The published sequence for *B. cereus* 4342 shows two T→C substitutions within the interrogated sequence, which may explain its larger increase in T_m_ and formation of its own outlying cluster.

**Figure 11:**
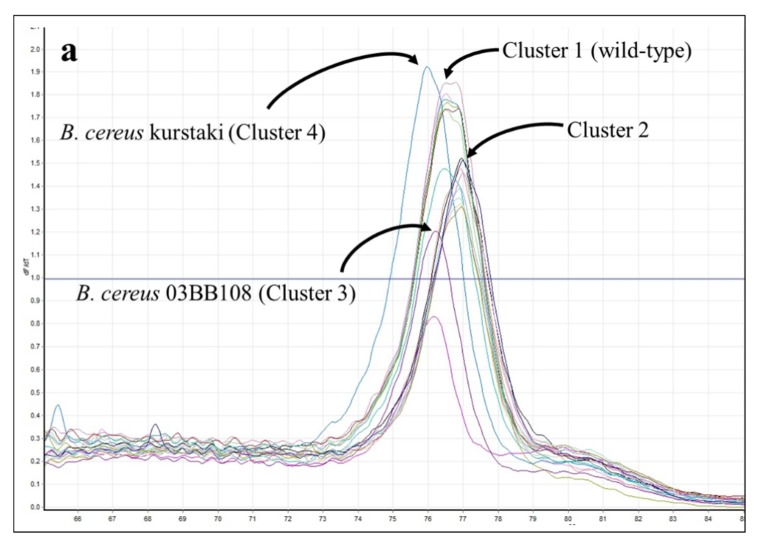

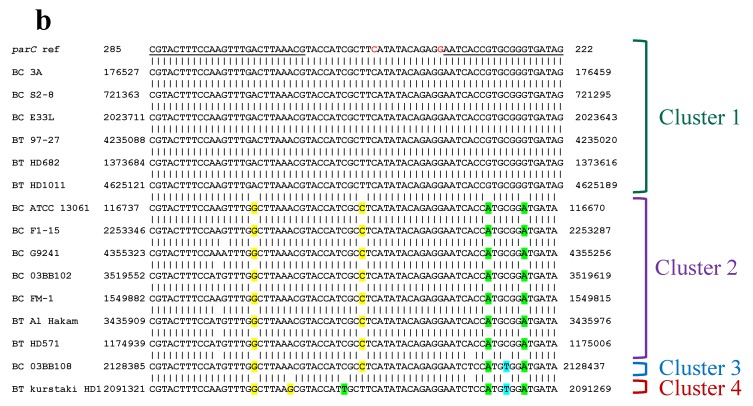
HRMA—*B. cereus* and *B. anthracis parC*. (**a**) Melt curves for *B. cereus* and *B. thuringiensis parC* amplicons. (**b**) Comparison of reference *B. anthracis parC* sequence with published sequences for the *B. cereus* (BC) and *B. thuringiensis* (BT) strains (see Table 13 for accession numbers). Primer sequences are underlined; mutations at the locations shown in red have been documented to cause fluoroquinolone resistance in *B. anthracis*. Substitutions predicted to increase or decrease amplicon T_m_s are highlighted in yellow or green, respectively.

In *parC* HRMA assays, roughly half of both *B. cereus* and *B. thuringiensis* strains clustered with wild-type *B. anthracis* controls (cluster 1) and most of the remainders formed a separate cluster (cluster 2). There were two outliers: *B. cereus* 03BB108 clustered with *B. anthracis* mutant S3-5 (cluster 3) and *B. thuringiensis* kurstaki formed its own cluster (cluster 4) (Table 8). Comparisons of published sequences (Figure 11) support our hypothesis that strains with sequences identical to the reference should—and do—cluster with *B. anthracis* wild-type, while those with a T→C change in the interrogated region should have higher T_m_s and cluster separately (cluster 2; Figure 11). Similarly, *B. thuringiensis* kurstaki, having a predicted C→T substitution, should produce an amplicon with a lower T_m_, which we observed here (cluster 4). It is unclear, however, why *B. cereus* 03BB108 (predicted T→C substitution) produced an amplicon with lower T_m_ and did not fall within cluster 2. For this strain—and all of these near neighbors—it is possible that various substitutions within the primed regions affected the melt curves in ways that could not always be predicted.

We did not have access to *Y. pestis* or *F. tularensis* strains with known mutations causing ciprofloxacin resistance in this study. Therefore, we were unable to assess whether HRMA could be used to discriminate between wild-type and resistant strains within these two genera. Fortunately, we were clearly able to distinguish between wild-type and mutant strains of *B. anthracis* in a readily observed and relatively predictable fashion.

Furthermore, in spite of numerous failures in *parC* amplifications, clustering of most near neighbor species for *gyrA*, *parC*, and *parE* in all three genera could be predicted through comparisons of the sequences targeted. *F. tularensis* could be easily discriminated from *F. philomiragia* based on *gyrA*, but not *parE* melt curves, potentially due to the short (5-nt) *parE* sequence targeted. Similarly, as expected, all *Y. pestis* and wild-type *B. anthracis* clustered with most, but not all, near neighbor strains having identical target sequences, though some anomalous clustering was observed (e.g., *Y. pseudotuberculosis* strains AMC TB4 and IP32983, B. *cereus* 03BB108). It is certainly possible that the unpredictable clustering may simply have been due to noisy data [43]. More likely, these anomalous results likely represent contaminants within the samples or errors introduced through use of whole genome amplicons in these HRMA studies. While φ29 DNA polymerase is much less prone to errors than other DNA polymerases, any errors introduced in the targeted QRDRs (or even potentially within the primer regions) could result in anomalous melt curves and subsequent clustering. A final possibility is that these anomalies may be due to spontaneous mutations or genetic drift. Assessment of this possibility would require additional amplicon sequencing, which was not performed here.

## 3. Materials and Methods

### 3.1. Materials

All genomic materials were obtained from Unified Culture Collection (UCC) of the Defense Biological Product Assurance Office (DBPAO), Frederick, MD USA and were provided as purified, certified inactive preparations previously extracted using Epicentre MasterPure DNA Purification kits (Lucigen Corporation, Middletown, WI, USA). *Francisella* spp., *Yersinia* spp., and *Bacillus* spp. strains used in this study are shown in Table 9, Table 10, Table 11, Table 12 and Table 13. Forty-six of the DNA preparations were derived from Select Agent Strains, as indicated.

### 3.2. Microarray Analysis Using the ARDM v.3.1

Illustra GenomiPhi HY kits (GE Healthcare, Pittsburgh, PA, USA) were used to amplify preparations of genomic DNA from each strain per the manufacturer’s instructions, using 10 ng of starting material. An amplification time of 90 min at 30 °C was used, followed by a 10 min enzyme inactivation step at 95 °C. After quantification using Qubit 2.0 fluorometer (Thermofisher, Rockland, IL, USA), amplicons were subjected a combined fragmentation/biotin-labeling step using the Bionick DNA-Labeling System (ThermoFisher); this method has proven superior to sequential fragmentation and biotinylation for subsequent microarray analysis on the ARDM [52]. Each reaction ─ sufficient for hybridization on two sub-arrays ─ used 3.2 µg whole genome amplicon in a final volume of 40 µL; reactions were incubated for 1–1.5 h at 16 °C before addition of 5 µL Stop Buffer. Twenty-two microliters of 3× hybridization buffer (18× SSPE/0.15% Tween-20/60 mM EDTA/300 ng/µL denatured salmon sperm DNA) was then added to each sample, samples were heated to 95 °C for 3 min, and cooled on ice for 3 min before loading on hydrated microarrays previously incubated for at least 30 min at 60 °C in prehybridization buffer (6× SSPE/0.05% Tween-20/20 mM EDTA/5×Denhardt’s solution/0.05% SDS/100 ng/µL denatured salmon sperm DNA; 30 µL per sub-array; Customarray, Bothell, WA, USA). After overnight hybridization at 60 °C, microarrays were washed twice at 60 °C in 3× SSPET (3× SSPE/0.05% Tween-20), twice at room temperature with 0.5× SSPET (0.5× SSPE/0.05% Tween-20), and twice at room temperature with PBST Wash (2× PBS/0.1% Tween-20). Arrays were then treated sequentially for 15 min with Biotin Blocking Solution (Customarray), and then 30 min with polymeric horseradish peroxidase-streptavidin conjugate (Cat. No. 65R-S104PHRP [Fitzgerald, Acton, MA, USA], diluted 1:1000 in PBS/10 mg/mL bovine serum albumin/0.05% Tween-20). Microarrays were then washed three times (5 min each wash) with PBST Wash Solution (Customarray) and finally interrogated as previously described [27,52].

The ARDM v.3.1 has a total of four subarrays (two pairs of identical subarrays, 2240 probes/subarray). The subarrays are based on two previous versions—ARDM v.2 (238 AMR determinants) [27,53,54], and ARDM v.3 (542 AMR determinants) [28]—and was re-designed to remove non-relevant or redundant content and emphasize AMR-related content from Select Agents. Each microarray slide could therefore analyze two samples (each sample = two subarrays). Each determinant on the microarray was represented by 8–10 probes, typically corresponding to 4–5 duplicate pairs of probes. Samples were called positive for a gene if signals from half of the gene’s probes exceeded the 95% threshold (mean signal from lowest 2128 probes + 3 standard deviations, SD) or if ≥70% of the probes had signals exceeding two lower stringency thresholds [29]. Using a combination of these thresholds has allowed us to detect families of closely related genes using probes with minor mismatches.

### 3.3. High Resolution Melt Analysis (HRMA)

The HRMA protocol, primers, and conditions used here were based on those described by Loveless [34] and are shown in Table 14. The analyses were performed using Type-it HRMA PCR kit (cat#206542, Qiagen, Germantown, MD, USA). The total volume of HRMA reactions was 25 µL and the reaction mixes were prepared according to the manufacturer instructions. Final concentrations of the primers (Eurofins/Operon, Louisville, KY, USA) in the reaction mix were 0.7 µM; template DNA was added to achieve the final concentration of approximately 100 pg/µL. The HRMA PCRs were performed on Rotor-Gene Q MDx (Qiagen, Hilden, Germany) using the following cycling conditions: 5 min at 95 °C, followed by 45 cycles of (10 s at 95 °C, 30 s at 55 °C), ending with melt curve generation (65 °C to 95 °C at 0.1 °C increments, 2 s each). Samples with C_t_ > 35 cycles or without sigmoidal amplification curves were called negative or invalid, respectively. Melt curves of valid amplifications were analyzed and clustering performed using ScreenClust software with unsupervised cluster analysis [55].

## 4. Conclusions

Both PCR and microarrays are valuable tools for the tracking the genetic underpinnings of AMR resistance. Here, we used two complementary technologies—microarray analysis and HRMA—to survey 127 Select Agents, exempt strains, and near-neighbor species for a broad variety of resistance mechanisms acquired through both horizontal transfer and gene mutations. To our knowledge, this is the largest survey of Category A agents, exempt strains, and near-neighbor species for genes covering multiple mechanisms of AMR.

Both of the methods used here had benefits and limitations. The broad detection capabilities of the microarray analysis enabled detection of the genes that we expected to find within the Select Agent species for all three genera. We further detected a non-native gene within a Select Agent strain (KIM (pCD1Ap)+) and detected families of similar genes in near-neighbor species; detection of these related genes was highly dependent on the phylogenetic distance (and hence, sequence conservation) between the near neighbor species and the species from which the reference sequence was derived. However, each microarray analysis is only as good as its content, and while its content is relatively comprehensive (500+ AMR genes), the ARDM v.3.1 is certainly not all-encompassing and is unable to detect small mutations that can affect specificity. HRMA, on the other hand, was able to rapidly discriminate between ciprofloxacin-resistant *B. anthracis* strains with point mutations within QRDRs of *gyrA* and *parC*, though several strains clustered anomalously (albeit separately from wild-type strains). One limitation of HRMA is that each reaction must be designed for each target sequence and reactions are not easily multiplexable. Furthermore, changes in DNA sequence detectable by HRMA do not always represent a change in amino acid sequence, and near-neighbor species may be erroneously identified as resistant mutants.

Provided suitable sensitivity can be achieved and data processing simplified, this is where rapidly evolving technologies such as next generation sequencing (NGS) may find tremendous application [56]. Several excellent studies have recently demonstrated use of targeted pyrosequencing and next generation sequencing approaches to rapid detect and identify both horizontally and vertically transmitted AMR determinants within Select Agent strains [57,58,59]. While these recent studies emphasized a limited number of AMR genes, we hope that these technologies will prove useful in performing similarly broad-based surveys of Select Agents and other pathogenic species.

## Figures and Tables

**Table 1:** Recommended therapeutic, prophylactic interventions for tularemia, plague, and anthrax.

	First Line Therapy ^1^	Alternative Therapy	Post-Exposure Prophylaxis
Tularemia	STR	GM or tetracycline family	CIP
Plague	STR or GM	CIP, DOX, or CHL	CIP or DOX
Anthrax	CIP + MEM + LZD	LVX (or MXF) + IPM (or DOR) + CLI (or CHL or RIF)	CIP or DOX

^1^ CHL = chloramphenicol; CIP = ciprofloxacin; CLI = clindamycin; DOR = doripenem; DOX = doxycycline; GM = gentamicin; IPM = imipenem; LVX = levofloxacin; LZD = linezolid; MXF = moxifloxacin; MEM = meropenem; RIF = rifampin; STR = streptomycin. Data compiled from [10,11,12].

**Table 2:** Microarray-detected antimicrobial resistance (AMR) genes in *Francisella* spp. strains.

Strain	Resistance Determinants by Class		
	β-Lactams	Tetracyclines	Multidrug Efflux Pumps
***F. philomiragia* (*n* = 3)**			
Jensen O#319L, Jensen O#319-036	*bla* ^1^		
Jensen O#319-029	*bla* ^1^		*bcr/cflA1*
***F. tularensis* (*n* = 20)**			
NIH B-38, U112, LVS, DS89-R-54, DS88-R-675, DS88-R-160, DS87-R-135, DS88-R-144, DS87-R-200, DST6755, DSAL91-1623, DS88-R-147, SCHU-S4, JAP (Cincinnati), VT68, strain 425, Scherm, WY96-3418, GA99-3548, GA99-3549	*blaA*, *blaB*	*tet*	*bcr/cflA1*, *bcr/cflA2*, *bcr/cflA3*

^1^ F. philomiragia-derived bla gene.

**Table 3:** Microarray-detected AMR genes in *Yersinia* spp. strains.

UCC Strain	Resistance Determinants by Class				
	β-Lactams	Baci-tracin	Quater-nary amines	Strepto-gramins	Multidrug Efflux Pumps
***Y. pestis* (*n* = 17)**					
Antigua, Nairobi, PBM19, Pestoides, Pestoides F, Harbin35, Java9, CO92, KIM 10v, Dodson, Shasta, A1122, Pestoides G, Angola, Nicholisk 41, El Dorado 2572-1		*bacA*	*qacE*		*emrA*, *emrB*, *yegB*
KIM (pCD1Ap)+	*bla*_TEM_	*bacA*	*qacE*		*emrA*, *emrB*, *yegB*
***Y. pseudotuberculosis* (*n* = 9)**					
AMC TB4, ATCC 4284, ATCC 6904, IP32953, YPIII, Pa3606, EP2/+, MD67, strain 1		*bacA*	*qacE*		*emrA*, *emrB*, *yegB*
***Y. enterocolitica* (*n* = 7)**					
ATCC 33114, ATCC 55075, E265, DATR, WA, 2516-87				*vatF/sat*	
8081	(none)				
**Other *Yersinia* spp. (*n* = 9)**					
*Y. aldovae* 670-83 *Y. frederiksenii* 1461-81 and Y225 *Y. intermedia* Y228 *Y. kristensenii* ATCC 33639 and Y231 *Y. rohdei* ATCC 43380 *Y. ruckeri* ATCC 29473 and YERS063	(none)				

**Table 4:** Microarray-detected AMR genes in *B. cereus* group strains.

UCC Strain	Resistance Determinants by Class						
	β-Lactams	Aminoglycosides	Macro-lides	Tetra-cyclines	Glyco-peptides	Phenicols	Fosfo-mycin
***B. anthracis* (*n* = 41)**							
V770-NP1-R, Ames, Smith 1013, Delta Sterne, Vollum 1B, Zimbabwe 89, Pakistan SK-102, Scotland K1811, BA0052, Canadian Bison, Pasteur, RA3, strain 108, Ohio ACB, Turkey 32, A0435, 2000031021, 2002013094, ΔANR, S1-1, S2-1, S2-3, S3-3, S3-4, S3-5, S3-6, S3-7, S3-8, S3-11, S3-12, S3-17, S3-18	*bla1*, *bla2* *bla*[Vollum1] *bla*[Vollum3] *bla*[Vollum4]	*aacC7, aad*[Vollum1] *aad*[Vollum2] *aph*[Vollum1] *aph*[Vollum2] *aph*[Vollum3]	*mph*	*tetM*	*vanY*	*cat*	*fosB*
Vollum, Sterne, South Africa, S1-2, S2-2, S3-1, S3-14, S3-15, S3-16	*bla1*, *bla2* *bla*[Vollum1] *bla*[Vollum3] *bla*[Vollum4]	*aacC7, aad*[Vollum1] *aad*[Vollum2] *aph*[Vollum1] *aph*[Vollum2] *aph*[Vollum3]	*mph*	*tetM*	*vanY*	*cat* *cap*(pSCS5)	*fosB*
***B. cereus* (*n* = 13)**							
NRRLB-569	*bla1*, *bla2* *bla*[Vollum3] *bla*[Vollum4]	*aad*[Vollum1] *aph*[Vollum1] *aph*[Vollum3]		*tetM*	*vanY*		
NRL 731	*bla*[Vollum1] *bla*[Vollum3] *bla*[Vollum4]	*aad*[Vollum1] *aad*[Vollum2] *aph*[Vollum1] *aph*[Vollum3]				*cat*	*fosB*
PCI 246	*bla2*, *bla*[Vollum1] *bla*[Vollum3] *bla*[Vollum4]	*aacC7, aad*[Vollum1] *aph*[Vollum1] *aph*[Vollum3]		*tetM*			*fosB*
F1-15	*bla1*, *bla2* *bla*[Vollum1] *bla*[Vollum3] *bla*[Vollum4]	*aad*[Vollum1] *aph*[Vollum1] *aph*[Vollum3]		*tetM*	*vanY*		*fosB*
G9241, FM-1	*bla1*, *bla2* *bla*[Vollum1] *bla*[Vollum3] *bla*[Vollum4]	*aad*[Vollum1] *aad*[Vollum2] *aph*[Vollum1] *aph*[Vollum2] *aph*[Vollum3]		*tetM*		*cat*	*fosB*
4342	*bla1*, *bla2*, *bla*[Vollum1] *bla*[Vollum3] *bla*[Vollum4]	*aacC7, aad*[Vollum1] *aad*[Vollum2] *aph*[Vollum1] *aph*[Vollum2] *aph*[Vollum3]			*vanY*	*cat*	*fosB*
D17	*bla2* *bla*[Vollum1] *bla*[Vollum3] *bla*[Vollum4]	*aacC7, aad*[Vollum1] *aad*[Vollum2] *aph*[Vollum1] *aph*[Vollum2] *aph*[Vollum3]	*mph*	*tetM*	*vanY*	*cat*	*fosB*
03BB108	*bla1*, *bla2* *bla*[Vollum3] *bla*[Vollum4]	*aacC7, aad*[Vollum1] *aad*[Vollum2] *aph*[Vollum1] *aph*[Vollum2] *aph*[Vollum3]	*mph*	*tetM*	*vanY*	*cat*	*fosB*
3A, 03BB102, E33L, S2-8	*bla1*, *bla2* *bla*[Vollum1] *bla*[Vollum3] *bla*[Vollum4]	*aacC7, aad*[Vollum1] *aad*[Vollum2] *aph*[Vollum1] *aph*[Vollum2] *aph*[Vollum3]	*mph*	*tetM*	*vanY*	*cat*	*fosB*
***B. thuringiensis* (*n* = 8)**							
var israelensis	*bla*[Vollum3]	*aph*[Vollum3]					
morrisoni	*bla*[Vollum3]	*aph*[Vollum3]				*cat*	
kurstaki	*bla1*, *bla2*, *bla*[Vollum3]	*aad*[Vollum1] *aph*[Vollum1]		*tetM*		*cat*	
Al Hakam, 97-27, HD1011, HD682, HD571	*bla1*, *bla2*, *bla*[Vollum1], *bla*[Vollum3], *bla*[Vollum4]	*aacC7, aad*[Vollum1] *aad*[Vollum2] *aph*[Vollum1] *aph*[Vollum2] *aph*[Vollum3]	*mph*	*tetM*	*vanY*	*cat*	*fosB*

**Table 5:** High resolution melt analysis (HRMA) melt temperatures and clusters for *Francisella* spp. strains.

Strain	*gyrA*		*parE*	
	T_m_	Cluster	T_m_	Cluster
*F. tularensis*				
NIH B-38	74.92	1	73.00	1
DS87-R-135	74.95	1	73.02	1
DS88-R-144	74.9	1	73.00	1
JAP (Cincinnati)	74.95	1	invalid ^1^	-
WY96-3418	74.87	1	73.02	1
GA99-3548	74.9	1	73.05	1
GA99-3549	74.93	1	73.03	1
*F. philomiragia*				
Jensen O#319L	75.98	2	72.83	1
Jensen O#319-029	75.67	2	72.90	1
Jensen O#319-036	76.03	2	73.00, 73.80	1

^1^ Did not have characteristic sigmoidal amplification curves.

**Table 6:** *GyrA* melt temperatures and clusters for *Yersinia* spp. strains analyzed by HRMA.

Sample	T_m_^1^	Cluster	Sample	T_m_	Cluster
*Y. pestis*					
PBM19	79.87, 80.15	1	Shasta	79.85, 80.1	1
Pestoides F	79.85, 80.2	1	A1122	79.88	1
Java9	79.82, 80.22	1	Pestoides G	79.83, 80.22	1
KIM 10v	79.85, 80.18	1	KIM (pCD1Ap)+	79.8, 80.2	1
Dodson	79.83, 80.17	1	El Dorado 2572-1	79.1, 79.83, 80.17	1
*Y. pseudotuberculosis*					
AMC TB4	79.13, 79.98	2	Pa3606	79.88	1
ATCC 4284	79.9, 80.17	1	EP2/+	79.92	1
IP32953	79.85	2	MD67	79.95	1
YPIII	79.9	1	strain 1	79.88	2
*Y. enterocolitica*					
ATCC 33114	79.9, 80.25	1	WA	79.9	2
ATCC 55075	79.85, 80.23	1	8081	79.95	3
E265	79.87, 80.18	1	2516-87	79.97	1
DATR	79.03, 79.87	2			
Other *Yersinia* spp.					
*Y. kristensenii* ATCC 33639	79.85, 80.23	1	*Y. kristensenii* Y231	79.9	2
*Y. ruckeri* ATCC 29473	79.95	1	*Y. frederiksenii* Y225	79.88	1
*Y. rohdei* ATCC 43380	79.93	1	*Y. aldovae* 670-83	79.85	1
*Y. ruckeri* YERS063	79.9	2	*Y. intermedia* Y228	79.9	2

^1^ Some samples had multiple T_m_ values (determined by RotorGene Q series software), based on presence of multiple peaks on melt curves.

**Table 7:** HRMA melt temperatures and clusters for *B. anthracis* strains (wild-type and mutant).

Strain	*gyrA*			*parC*		
	Genotype	T_m_	Cluster	Genotype	T_m_	Cluster
V770-NP1-R	Wt ^1^	76.18	1	wt	76.48, 76.77	1
Vollum	wt	76.2	1	wt	invalid ^2^	-
Sterne	wt	76.15	1	wt	76.5, 76.85	1
Smith 1013	wt	76.13	1	wt	76.45	1
Delta Sterne	wt	76.15	1	wt	76.53	1
Zimbabwe 89	wt	76.17	1	wt	invalid	-
Pakistan SK-102	wt	76.2	1	wt	76.45	1
Scotland K1811	wt	76.2	1	wt	76.57	1
BA0052	wt	76.08	1	wt	76.55	1
Pasteur	wt	Invalid ^2^	-	wt	76.52	1
RA3	wt	76.15	1	wt	76.52	1
Strain 108	wt	76.03	1	wt	76.55	1
Ohio ACB	wt	76.15	1	wt	76.55	1
Turkey 32	wt	76.18	1	wt	76.53	1
A0435	wt	76.12	1	wt	76.5	1
2000031021	wt	76.13	1	wt	76.45	1
2002013094	wt	76.05	1	wt	76.5	1
∆ANR	wt	76.15	1	wt	76.63	1
S1-1	C254→T ^4^	75.43	2	wt	75.43	2A ^3^
S1-2	G265→A	75.43	2	wt	76.42	3A
S2-1	C254→T	75.45	2	C242→T	76.45	2A
S2-2	C254→T	75.43	2	C242→A	76.02	2A
S2-3	C254→T	75.45	2	C242→A or G253→A	75.9	2A
S3-1	C254→T	75.45	2	C242→T	75.92	2A
S3-3		75.42	2		75.85	2A
S3-4		75.42	2		75.9	2A
S3-5		74.75	3		76.1	2B
S3-6	C254→T	75.5	2	C242→T	76.18	2B
S3-7		75.5	2		76.18	2B
S3-8	C254→T	75.52	2	C242→T	76.12	2B
S3-11	C254→T; G265→A	75.52	2	C242→T	invalid	-
S3-12		75.5	2		invalid	-
S3-14		75.5	2		invalid	-
S3-15		74.72	3		invalid	-
S3-16	C254→T; A266→C	75.5	2	C242→T	76.12	2B
S3-17		75.52	2		76.1	2B
S3-18		75.5	2		invalid	-

^1^ Presumptive wild-type (genotypic confirmation not performed). ^2^ Samples without characteristic sigmoidal amplification curves were deemed invalid. ^3^ Two clusters separate from wild-type were observed on Day 1 (2A, 3A), whereas only a single non-wild-type cluster was observed on Day 2 (2B). ^4^ Substitutions shown were described by [6] or [34].

**Table 8:** HRMA melt temperatures and clusters for *B. cereus* and *B. thuringiensis* strains.

	*gyrA*		*parC*	
Strain	T_m_	Cluster	T_m_	Cluster
	*B. cereus*			
NRRLB-569	76.9	2	invalid	-
PCI 246	75.95	1	76.93	2
F1-15	invalid	-	76.95	2
3A	75.92	1	76.47	1
G9241	75.9	1	76.87	2
03BB102	75.9	1	76.9	2
D17	75.95	1	invalid	-
4342	77.45	3	77	2
E33L	76.5, 76.92	2	76.55, 76.8	1
S2-8	75.93	1	76.5	1
FM-1	75.93	1	77	2
03BB108	75.9	1	76.22	3
	*B. thuringiensis*			
var israelensis	76.92	2	invalid	-
kurstaki	76.92	2	76.17	4
Al Hakam	75.95	1	76.92	2
97-27	75.95	1	76.5	1
HD1011	75.93	1	76.52, 76.78	1
HD682	75.9	1	76.53, 76.85	1
HD571	75.9	1	76.95	2
morrisoni	76.95	2	invalid	-

**Table 9:** *Francisella* spp. strains used in this study.

Strain Designation, Description	Alternative Strain Designation	DBPAO/UCC Strain No.	NCBI Accession Number(s)	Reference
*F. tularensis*				
NIH B-38	ATCC 6223	FRAN001	CP010115, KN046815	[44]
U112	ATCC 15482; CDC GA99-3550	FRAN 003	CP009633	[44]
LVS	LVS (var. palearctica)	FRAN004	CP009694	[44]
DS89-R-54 *		FRAN005		
DS88-R-675 *		FRAN006		
DS88-R-160 *		FRAN007	KN046806; JPGP00000000	[45]
DS87-R-135 *		FRAN008	JOOU00000000	[45]
DS88-R-144 *		FRAN009	JOOV00000000	[45]
DS87-R-200 *		FRAN 010	KN046802	[45]
DST6755 *		FRAN 011	KN046796-KN046797; JOUF00000000	[45]
DSAL91-1623 *		FRAN 012		
DS88-R-147 *		FRAN 015	KN046810; JOOR00000000	[45]
SCHU-S4 *		FRAN016	CP010290	[44]
JAP (Cincinnati) *		FRAN024	JUJU00000000	[44]
VT68*		FRAN025	CP010288	[44]
strain 425 *		FRAN029	CP010289	[44]
Scherm *		FRAN031	CP010287	[44]
WY96-3418 *		FRAN072	CP010103	[44]
GA99-3548		FRAN133	NZ_DS264587-NZ_DS264591	
GA99-3549		FRAN134	DS264124-DS264132	
*F. philomiragia*				
Jensen O#319L	ATCC 25015	FRAN002	CP010019	[44]
Jensen O#319-029	ATCC 25016	FRAN017	CP009342–CP009343	[44]
Jensen O#319-036	ATCC 25017	FRAN018	CP009442–CP009443	[44]

* Select Agent strain.

**Table 10:** *Y. pestis* strains used in this study.

Strain Designation, Description	Alternative Strain Designation	DBPAO/UCC Strain No.	NCBI Accession Number(s)	Ref.
Antigua *		YERS016	CP009903– CP009906	[46]
Nairobi	YE0237; WRAIR 710	YERS017	CP010293– CP010294	[46]
PBM19 *		YERS 018	CP009489–CP009492	[46]
Pestoides *		YERS 019	CP010020–CP010023	[46]
Pestoides F *		YERS020	CP009713–CP009715	[46]
Harbin35		YERS021	CP009701–CP009704	[46]
Java9 *		YERS022	CP009992–CP009996	[46]
CO92*		YERS023	JPMB00000000; KN150724-KN150730	[47]
KIM 10v *		YERS061	LFXP01000001-LFXP01000006	[48]
Dodson *		YERS073	CP009842–CP009844	[46]
Shasta *		YERS074	CP009721–CP009724	[46]
A1122	ATCC 11953; WRAIR 720	YERS078	CP009839–CP009841	[46]
Pestoides G *		YERS079	CP010246–CP010248	[46]
Angola *	NCBI39746	YERS080	CP009934–CP009937	[46]
KIM (pCD1Ap)+ *		YERS082		[31]
Nicholisk 41		YERS083	CP009988– CP009991	[46]
El Dorado 2572-1 *		YERS100	CP009782– CP009785	[46]

* Select Agent strain.

**Table 11:** Other *Yersinia* spp. strains used in this study.

Strain Designation, Description	Alternative Strain Designation	DBPAO/UCC Strain No.	NCBI Accession Number(s)	Ref.
*Y. enterocolitica* (*n* = 7)				
ATCC 33114	ATCC 9610; CCUG 11291; CCUG 12369; CIP 80.27; DSM 4780; LMG 7899; NCTC 12982	YERS001	JPDV00000000; KN150735	[47]
ATCC 55075		YERS004		
E265	YE1012	YERS014	JPDW00000000; KN150736-KN150738	[47]
DATR	YE1013	YERS015	JPDU00000000; KN150733-KN150734	[47]
WA		YERS093	CP009366, CP009367	[46]
8081		YERS094	CP009845, CP009846	[46]
2516-87		YERS095	CP009837, CP009838	[46]
*Y. pseudotuberculosis* (*n* = 9)				
AMC TB4	ATCC 6904; YE1022	YERS008		
ATCC 4284	MIDI 3293	YERS036	JPIY00000000; KN150741-KN150744	[47]
ATCC 6904	NCTC 2476	YERS038	CP008943	[47]
IP32953		YERS086	CP009710– CP009712	[46]
YPIII		YERS087	CP009792	[46]
Pa3606		YERS088	CP010067– CP010069	[46]
EP2/+		YERS090	CP009758, CP009759	[46]
MD67		YERS091	CP009757	[46]
strain 1		YERS092	CP009786	[46]
Misc. *Yersinia* spp. (*n* = 9)				
*Y. aldovae* 670-83		YERS098	CP009781	[46]
*Y. frederiksenii* CDC 1461-81	ATCC 33641; CIP 80-29; YE1056	YERS005	JPPS00000000; KN150731-KN150732	[47]
*Y. frederiksenii* Y225		YERS097	CP009363, CP009364	[46]
*Y. intermedia* Y228		YERS099	CP009801	[46]
*Y. kristensenii* ATCC 33639	CDC 1459-81	YERS002	CP008955	[47]
*Y. kristensenii* Y231		YERS096	CP009997	[46]
*Y. rohdei* ATCC 43380	CDC 3022-85	YERS062	CP009787	[46]
*Y. ruckeri* ATCC 29473	CDC 2396-61; NCIMB 2194	YERS012	JPPT00000000; KN150747-KN150748	[47]
*Y. ruckeri* YERS063		YERS063		

**Table 12:** *B. anthracis* strains used in this study.

Strain Designation, Description	Alternative Strain Designation	DBPAO/UCC Strain No.	NCBI Accession Number(s)	Ref
	*B. anthracis* (*n* = 41)			
V770-NP1-R		BACI002	CP009597- CP009598	[49]
Vollum*		BACI007	CP007664-CP007666	[50]
Ames*		BACI008	CP009979-CP009981	[49]
Sterne		BACI012	CP009540-CP009541	[49]
Smith 1013 *		BACI055		
Delta Sterne		BACI056	CP008752	[50]
Vollum 1B *	USAMRIID BA1002	BACI124	CP009326-CP009328	[49]
Zimbabwe 89 *		BACI125	KN050648-KN050650	[50]
Pakistan SK-102 *		BACI126	CP009462- CP009464	[49]
Scotland K1811 *		BACI128		
BA0052 *		BACI131	CP007702-CP007704	[50]
Canadian Bison *	CDC 607; USAMRIID BA0018	BACI153	CP010320-CP010322	[49]
Pasteur *		BACI155	CP009475-CP009476	[49]
South Africa *	BA1035	BACI207	CP009698-CP009700	[49]
RA3 *		BACI225	CP009695-CP009697	[49]
strain 108 *	USAMRIID BA1015	BAC226		[49]
Ohio ACB *		BACI259	CP009339-CP009341	[49]
Turkey 32 *	A0149	BACI260	CP009314-CP009316	[49]
A0435 *	K3	BACI261	CP009329-CP009331	[49]
2000031021 *		BACI292	CP007617-CP007618	[50]
2002013094 *		BACI293	CP009900-CP009902	[49]
ΔANR		BACI355		[6]
S1-1		BACI356		[6]
S1-2		BACI357		[6]
S2-1		BACI358		[6]
S2-2		BACI359		[6]
S2-3		BACI360		[6]
S3-1		BACI361		[6]
S3-3		BACI362		[6]
S3-4		BACI363		[6]
S3-5		BACI364		[6]
S3-6		BACI365		[6]
S3-7		BACI366		[6]
S3-8		BACI367		[6]
S3-11		BACI368		[6]
S3-12		BACI369		[6]
S3-14		BACI370		[6]
S3-15		BACI371		[6]
S3-16		BACI372		[6]
S3-17		BACI373		[6]
S3-18		BACI374		[6]

* Select Agent strain.

**Table 13:** *B. cereus* and *B. thuringiensis* strains used in this study.

Strain Designation, Description	Alternative Strain Designation	DBPAO/UCC Strain No.	NCBI Accession Number(s)	Ref
*B. cereus* (*n* = 13)				
NRRLB-569	ATCC 10876; NRRL B-569; NRS 1256	BACI015	KN050654, KN050655; JMPW01000022	[50]
PCI 246	ATCC 10876a; ATCC 13061; ATCC 13640; IFO 13494; NCTC 9946; NRRL B-3537	BACI016	JMPX01000039-JMPX01000053	[50]
NRL 731	ATCC 4342; MIDI 3561; NRS 731	BACI083	JMPW01000022	[50]
F1-15		BACI227	NZ_KN049962-NZ_KN049966	[50]
3A		BACI228	CP009593–CP009596	[49]
G9241		BACI232	CP009589–CP009592	[49]
03BB102		BACI234	CP009317–CP009318	[49]
D17		BACI262	CP009299–CP009300	[49]
4342		BACI263	CP009627–CP009628	[49]
E33L	Zebra killer	BACI267	CP009965–CP009970	[49]
S2-8		BACI268	CP009604–CP009606	[49]
FM-1		BACI290	CP009368–CP009369	[49]
03BB108		BACI291	CP009634–CP009641	[49]
*B. thuringiensis* (*n* = 8)				
var. israelensis	ATCC 35646; USDA HD522	BACI036		
kurstaki	ATCC 33679; NRRL B-3792	BACI204	CP009998–CP010012	[49]
Al Hakam		BACI229	NC_008600, CP000485	[51]
97-27		BACI230	CP010087–CP010088	[49]
HD1011		BACI264	CP009332–CP009336	[49]
HD682		BACI265	CP009717–CP009720	[49]
HD571		BACI266	CP009599–CP009600	[49]
morrisoni	NRRL HD-597	BACI289	JTHH01000001- JTHH01000008	[49]

**Table 14:** Primers, probes for HRMA, derived from Loveless [34].

Species	Primers
*F. tularensis*	gyrA-F: TCGGTAAATATCACCCTCATGGAG gyrA-R: AGGTTGTGCCATTCTGACAATAGTAT
	parE-F: CTTACATGGCATTTTGAAACTGGAC parE-R: CAGCTTCTAGTTTATGGTCAAGATAGCC
*Y. pestis*	gyrA-F: ATCGGTAAATACCACCCGCAT gyrA-R: CGCACGATAGTGTCGTAGACCG
*B. anthracis*	gyrA-F: GGTAAGTATCACCCTCATGGTGATT gyrA-R: GCGCCATACGTACCATCGTT
	parC-F: CTATCACCCGCACGGTGATT parC-R: CGTACTTTCCAAGTTTGACTTAAACG

## Abbreviations

AMR	Antimicrobial resistance
ARDM	Antimicrobial Resistance Determinant Microarray
AST	Antimicrobial susceptibility testing
BC	*Bacillus cereus*
BT	*Bacillus thuringiensis*
CHL	Chloramphenicol
CIP	Ciprofloxacin
CLI	Clindamycin
DOR	Doripenem
DOX	Doxycycline
GM	Gentamicin
HRMA	High resolution melt analysis
IPM	Imipenem
LVX	Levofloxacin
LZD	Linezolid
MDR	Multidrug resistance
MEM	Meropenem
MXF	Moxifloxacin
QRDR	Quinolone resistance-determining region
RIF	Rifampin
STR	Streptomycin
